# Perceived Vulnerability to Disease and the Relationship with Teacher Satisfaction in South Africa during COVID-19: The Serial Role of Burnout, Role Conflict, and Ambiguity

**DOI:** 10.3390/bs12060160

**Published:** 2022-05-24

**Authors:** Tyrone Brian Pretorius, Anita Padmanabhanunni, Serena Ann Isaacs, Kyle Jackson

**Affiliations:** Department of Psychology, University of the Western Cape, Bellville 7530, South Africa; apadmana@uwc.ac.za (A.P.); sisaacs@uwc.ac.za (S.A.I.); kmjackson@uwc.ac.za (K.J.)

**Keywords:** COVID-19, burnout, role ambiguity, role conflict, teacher satisfaction

## Abstract

Teachers’ work roles and responsibilities have changed dramatically because of the COVID-19 pandemic. These unprecedented changes have the potential to generate role stress and burnout and reduce teachers’ job satisfaction. This study investigated the serial relationship between perceived vulnerability to disease, role stress, burnout, and teaching satisfaction. It was hypothesised that individuals who perceive themselves to be at high risk of contracting COVID-19 would report high role conflict and ambiguity in the workplace, which would in turn lead to high levels of burnout and low satisfaction with teaching. Participants were schoolteachers (N = 355) who completed the Perceived Vulnerability to Disease Questionnaire, the Role Orientation Questionnaire, the Maslach Burnout Inventory, and the Teaching Satisfaction Scale. Path analysis confirmed that perceived vulnerability to disease was associated with role conflict and ambiguity, which was in turn associated with emotional exhaustion, depersonalisation, and low teaching satisfaction. Teachers who appraised themselves as being more vulnerable to contracting COVID-19 experienced greater role stress, which was associated with high levels of burnout and low teaching satisfaction. This study highlights that threat appraisals related to contracting COVID-19 represent an additional job demand and this needs to be matched by job resources that can facilitate coping.

## 1. Introduction

The COVID-19 pandemic precipitated unprecedented disruptions to the educational sector globally and led to nationwide school closures to curtail the transmission of the virus. Conventional classroom-based schooling ceased in many countries, and schools were required to transition to emergency remote learning or adopt hybrid approaches to education [1]. Teachers’ work roles and responsibilities changed dramatically as they were required to rapidly upskill their use of information communication technology, adapt lesson plans to suit online delivery, provide task differentiation, guide students and their parents to negotiate the online environment, and promote effective student engagement and learning [2,3]. These disruptions to the educational sector have significantly increased the job demands on teachers and may have generated role stress, which can present as role ambiguity and role conflict [4].

Role ambiguity is defined as a sense of uncertainty about which tasks and responsibilities are associated with a specific work role. This phenomenon can occur when work responsibilities are not clearly defined or when there is insufficient information available to accomplish one’s work role effectively [5]. Role conflict refers to incompatible or inconsistent demands. This phenomenon entails the simultaneous occurrence of two or more job pressures such that compliance with one makes it difficult to accomplish the other [5]. Role stress tends to be construed as a negative experience; however, it can lead to beneficial outcomes, such as increased administrative flexibility, adaptability to changing circumstances, and creative problem solving [6]. Nevertheless, a significant body of existing research has confirmed that both role conflict and role ambiguity are salient sources of job dissatisfaction and burnout e.g., [7,8,9,10]. 

The COVID-19 pandemic has added another dimension to teachers’ workload, which contributed to high levels of professional burnout even prior to the pandemic. Burnout is a psychological syndrome characterised by emotional exhaustion, depersonalisation, and reduced professional accomplishment [11]. It has been associated with impaired teacher functioning and reduced job satisfaction [12]. Teachers who experience burnout are more vulnerable than their peers to depression, anxiety, and sleep disturbances, and the quality of instruction they provide to learners is low. Teachers who experience burnout are also more likely than their peers to leave the profession and/or to retire early [12]. In the context of the current pandemic, fear of COVID-19 may have added another dimension of job stress for teachers and increased their vulnerability to burnout. As schools re-open in many countries, teachers may experience increasing anxiety about being exposed to the virus in the school environment and potentially transmitting it to their loved ones. These fears may be aggravated in circumstances in which personal protective equipment is not readily accessible and school infrastructure is not conducive to social distancing [13]. One of the factors influencing fear of COVID-19 is perceived susceptibility to infection and germ aversion, which entails personal beliefs or threat appraisals about the likelihood of contracting a disease and personal discomfort with and avoidance of situations that might increase exposure to disease [14,15,16,17,18]. The majority of studies investigating the role of threat appraisals and germ aversion during the pandemic have focused on its role in promoting adherence to COVID-19 safety protocols, e.g., [14,15]. Few studies have conceptualised these factors as a potential source of occupational distress that may contribute to adverse mental health outcomes, including burnout. 

The current study is framed within the Job-Demands and Resources model (JDR [19]), which has been consistently used as a theoretical framework to conceptualise and understand the influence of various types of stressors on teacher burnout, motivation, and job satisfaction. According to the JDR model, there are two characteristics associated with all occupations, namely, job demands and job resources. Job demands refer to the features of an occupation (e.g., roles and responsibilities) that require constant effort and are potentially associated with physical, emotional, or psychological costs. Job resources, on the other hand, entail the social, physical, and organisational aspects of an occupation that help the individual to achieve work-related tasks and goals, reduce job demands, and facilitate personal growth [19]. Previous research has investigated the influence of various job demands in the teaching profession including high workloads, low motivation levels among students, absence of administrative support, discipline issues with students, and conflict with colleagues [20,21,22]. In addition, studies [20,21,22] have also identified job resources including opportunities for growth and advancement, classroom management self-efficacy, and positive social relationships with colleagues. These job resources have been associated with increased work engagement, improved wellbeing, and greater job satisfaction among teachers. The JDR model has been extensively cited in the literature on occupational stress [20,21,22] and proposes that when job demands outweigh the resources available to cope, it may lead to burnout. 

This study was conducted in South Africa, where the COVID-19 pandemic and related preventive measures amplified existing inequalities in the educational sector. In March 2020, the South African government implemented a hard lockdown that included the closure of all schools and universities and a mandated transition to remote learning [23,24]. Due to historical factors, the schooling system in the country is sharply split along socioeconomic lines, and most schools were ill-equipped for the transition to online learning and teaching [13]. In addition, most South African households did not have internet access, and many teachers and parents were unable to afford the data needed to sustain remote learning [23]. Students lost an estimated 22–65% of their normal school days due to the transition to online learning [23,24]. 

Due to the unsustainability of remote teaching, conventional classroom-based teaching resumed in February 2021 in the context of the second and third waves of the pandemic [13]. Preliminary findings suggest that teachers experienced heightened levels of distress due to their perceived increase in vulnerability to infection and the lack of resources in many schools to effectively implement COVID-19 safety protocols [13]. In sum, the COVID-19 pandemic has placed South African teachers in a precarious position. Vaccine rollout for teachers was implemented on 23 June 2021; however, vaccine hesitancy was high. The most common reasons for vaccine hesitancy include concerns about its safety, negative attitudes towards vaccines, religious beliefs, and lack of knowledge and awareness [25]. The ensuing tension between fear of COVID-19 (i.e., becoming ill or making others ill), job satisfaction, role orientation, and burnout could decrease teachers’ job satisfaction, increase burnout, and ultimately lead to teacher attrition [26]. 

The aim of the current study was to investigate the serial relationship between perceived vulnerability to disease, role stress (i.e., role conflict and ambiguity), burnout, and teaching satisfaction. Our research questions focused on (i) whether teachers who perceived themselves as more vulnerable to infection reported greater perceptions of role conflict and ambiguity in the workplace, and (ii) if this, in turn, was associated with high levels of burnout and low levels of satisfaction with teaching.

We proposed the following research hypotheses:

**Hypothesis** **1** **(H1).** *Germ aversion (i.e., personal discomfort with and avoidance of situations that might increase exposure to disease), a component of perceived vulnerability to disease, will be associated with increased role ambiguity, which in turn will be associated with higher levels of emotional exhaustion.*

**Hypothesis** **2** **(H2).** *Germ aversion will be associated with increased role ambiguity, which in turn will be associated with higher levels of depersonalisation.*

**Hypothesis** **3** **(H3).** *Germ aversion will be associated with increased role ambiguity, which in turn will be associated with lower levels of personal accomplishment.*

**Hypothesis** **4** **(H4).** *Perceived infectability (i.e., threat appraisals about the likelihood of contracting a disease), a component of perceived vulnerability to disease, will be associated with increased role ambiguity, which in turn will be associated with high levels of e. emotional exhaustion.*

**Hypothesis** **5** **(H5).** *Perceived infectability will be associated with increased role ambiguity, which in turn will be associated with high levels of depersonalisation.*

**Hypothesis** **6** **(H6).** *Perceived infectability will be associated with increased role ambiguity, which in turn will be associated with low levels of personal accomplishment.*

**Hypothesis** **7** **(H7).** *Germ aversion will be associated with increased role conflict, which in turn will be associated with high levels of emotional exhaustion.*

**Hypothesis** **8** **(H8).** *Germ aversion will be associated with increased role conflict, which in turn will be associated with high levels of depersonalisation.*

**Hypothesis** **9** **(H9).** *Germ aversion will be associated with increased role conflict, which in turn will be associated with low levels of personal accomplishment.*

**Hypothesis** **10** **(H10).** *Perceived infectability will be associated with increased role conflict, which in turn will be associated with high levels of emotional exhaustion.*

**Hypothesis** **11** **(H11).** *Perceived infectability will be associated with increased role conflict, which in turn will be associated with high levels of depersonalisation.*

**Hypothesis** **12** **(H12).** *Perceived infectability will be associated with increased role conflict, which in turn will be associated with low levels of personal accomplishment.*

**Hypothesis** **13** **(H13).** *Germ aversion will be associated with increased role ambiguity, which in turn will be associated with high levels of emotional exhaustion. High levels of emotional exhaustion will then be associated with low levels of teaching satisfaction.*

**Hypothesis** **14** **(H14).** *Germ aversion will be associated with increased role ambiguity, which in turn will be associated with high levels of depersonalisation. High levels of depersonalisation will then be associated with low levels of teaching satisfaction.*

**Hypothesis** **15** **(H15).** *Germ aversion will be associated with increased role ambiguity, which in turn will be associated with low levels of personal accomplishment. Low levels of personal accomplishment will then be associated with low levels of teaching satisfaction.*

**Hypothesis** **16** **(H16).** *Perceived infectability will be associated with increased role ambiguity, which in turn will be associated with high levels of emotional exhaustion. High levels of emotional exhaustion will then be associated with low levels of teaching satisfaction.*

**Hypothesis** **17** **(H17).** *Perceived infectability will be associated with increased role ambiguity, which in turn will be associated with high levels of depersonalisation. High levels of depersonalisation will then be associated with low levels of teaching satisfaction.*

**Hypothesis** **18** **(H18).** *Perceived infectability will be associated with increased role ambiguity, which in turn will be associated with low levels of personal accomplishment. Low levels of personal accomplishment will then be associated with low levels of teaching satisfaction.*

**Hypothesis** **19** **(H19).** *Germ aversion will be associated with increased role conflict, which in turn will be associated with high levels of emotional exhaustion. High levels of emotional exhaustion will then be associated with low levels of teaching satisfaction.*

**Hypothesis** **20** **(H20).** *Germ aversion will be associated with increased role conflict, which in turn will be associated with high levels of depersonalisation. High levels of depersonalisation will then be associated with low levels of teaching satisfaction.*

**Hypothesis** **21** **(H21).** *Germ aversion will be associated with increased role conflict, which in turn will be associated with low levels of personal accomplishment. Low levels of personal accomplishment will then be associated with low levels of teaching satisfaction.*

**Hypothesis** **22** **(H22).** *Perceived infectability will be associated with increased role conflict, which in turn will be associated with high levels of emotional exhaustion. High levels of emotional exhaustion will then be associated with low levels of teaching satisfaction.*

**Hypothesis** **23** **(H23).** *Perceived infectability will be associated with increased role conflict, which in turn will be associated with high levels of depersonalisation. High levels of depersonalisation will then be associated with low levels of teaching satisfaction.*

**Hypothesis** **24** **(H24).** *Perceived infectability will be associated with increased role conflict, which in turn will be associated with low levels of personal accomplishment. Low levels of personal accomplishment will then be associated with low levels of teaching satisfaction.*

## 2. Materials and Methods

### 2.1. Participants

Participants (N = 355) consisted of a convenience sample of schoolteachers from across South Africa, the majority of whom resided in the Western Cape Province (82.3%). The majority of the sample were women (76.6%), resided in an urban area (61.7%), and taught at the primary school level (61.1%). The mean age of the sample was 41.89 years (±12.42, range = 23–73), and the mean number of years in the teaching profession was 15.7 (±11.75, range = 1–48). Of the participant group, 63.9% indicated they had lost a family member to COVID-19, and 27% indicated that they had lost a colleague to COVID-19. 

### 2.2. Measures

Participants completed a brief demographic survey, the Perceived Vulnerability to Disease Questionnaire (PVD-Q; [27]), the Role Orientation Questionnaire [5], the Maslach Burnout Inventory (MBI; [11]), and the Teaching Satisfaction Scale (TSS; [24]). The PVD-Q was designed to assess beliefs about personal vulnerability to infectious diseases. Some studies have used the total score as an index of perceived vulnerability, e.g., [16]. However, Duncan and colleagues [27] demonstrated in a comprehensive psychometric analysis that the scale consists of two conceptually distinct subscales—germ aversion (GA) and perceived infectability (PI)—that are only moderately correlated. Further, these two subscales appear to differentially predict other variables. The GA subscale consists of 8 items that assess emotional discomfort in circumstances associated with a high potential for disease transmission. The PI subscale consists of 7 items that assess beliefs regarding one’s susceptibility to infectious diseases. Participants responded to the 15 items on a 7-point scale that ranged from “strongly disagree” (1) to “strongly agree” (7). Possible scores for the GA subscale range from 8 to 56, and a higher score reflects higher discomfort with the potential for disease transmission. Possible scores for the PI subscale range from 7 to 49, and a higher score reflects higher perceived infectability. Example items for each subscale include “If an illness is going around, I will get it” (PI) and “I prefer to wash my hands pretty soon after shaking someone’s hand” (GA). The authors of [27] provided evidence of the discriminant and convergent validity of the two subscales and reported internal consistency estimates of 0.87 and 0.74 for the PI and GA subscales, respectively. However, other studies have reported unsatisfactory reliability coefficients for the GA subscale ([17]: α = 0.56; [18]: α = 0.59).

The Role Orientation Questionnaire measures two distinct dimensions related to perceptions of work role: role conflict (RC) and role ambiguity (RA). The RC subscale consists of 8 items that measure the degree of dissonance experienced regarding one’s role expectations. The RA subscale consists of 6 items that measure one’s lack of clarity regarding role expectations. Participants respond to the 14 items on a 6-point Likert scale that ranges from “Definitely not true of my job” (1) to “Definitely true of my job” (6). High scores on the two scales indicate high levels of role conflict and ambiguity. Example items of each subscale include “I have to work on unnecessary things” (RC) and “I know exactly what is expected of me” (RA). The original development study of the scale reported reliability coefficients (Cronbach’s alpha) of 0.87 and 0.82 for role ambiguity and role conflict, respectively. More recent studies e.g., [28,29,30], have also reported satisfactory reliability coefficients, ranging from RC = 0.81, RA = 0.85 to RC = 0.92, RA = 0.91.

The MBI is one of the most widely used measures of burnout. It consists of 22 items that assess three dimensions of burnout: emotional exhaustion (EE), depersonalisation (DP), and personal accomplishment (PA). The EE subscale is regarded as the core component of burnout, and it measures feelings of tiredness, fatigue, and drained emotional energy resulting from one’s work experience. The DP subscale measures negative and indifferent feelings toward students and colleagues that are associated with feelings of callousness and cynicism. The PA subscale measures one’s sense of accomplishment and effectiveness related to work experience. High levels of EE and DP, as well as low levels of PA, are indicative of burnout. Participants respond to the 22 items on a 7-point scale that ranges from “Never” (0) to “Every day” (6). The original study that developed the scale [11] reported satisfactory estimates of internal consistency (Cronbach’s alpha) ranging from 0.69 to 0.92, as well as evidence of convergent and discriminant validity. A review of the reliabilities reported in selected studies that have used the MBI in educational settings [31] found that reliabilities in these studies ranged from 0.50 to 0.90, and the reliability of the DP subscale was consistently lower than those of the other two subscales.

The TSS is a 5-item measure of teaching satisfaction. Example items include “In most ways, being a teacher is close to my ideal” and “I am satisfied with being a teacher”. Participants respond to items on a 5-point Likert scale that ranges from “Strongly Disagree” (1) to “Strongly Agree” (5). Ho and Colleagues [28] reported a reliability coefficient of 0.77 and provided evidence of convergent and criterion-related validity. More recent applications of the scale have reported reliabilities of 0.78 [32] and 0.92 [33].

### 2.3. Procedure

An electronic version of the four scales and the demographic survey was developed using Google Forms. Online meetings were held with provincial education departments to explain the purpose of the study and invite officials to assist with distribution of the electronic link to the questionnaire via their professional networks. The link to the form was also distributed to schoolteachers via social media platforms such as teachers groups on Facebook from April to June 2021, in the second year of the COVID-19 pandemic. Facebook was used, owing to the ease of distribution via this platform, particularly in the context of the national lockdown. Since the start of the pandemic in December 2019, several mutations or variants of COVID-19 emerged. At the time of data collection (June–April 2021), the Delta variant of the COVID-19 virus had been detected in South Africa and the number of cases and deaths were increasing at a rapid pace. The Delta variant was first identified in India in December 2020 and was 50% more transmissible than other variants [34]. Thus, strict lockdown protocols were enforced by the South African government, and schools were closed for longer than one year (until 26 July 2021 [35]). 

### 2.4. Data Analysis

Descriptive statistics, reliabilities (alpha and omega), and the intercorrelations between variables were obtained using IBM SPSS Statistics for Windows (version 26; IBM Corp., Armonk, NY, USA). To examine the serial relationship between perceived vulnerability to disease, role conflict and ambiguity, burnout, and teaching satisfaction, IBM SPSS Amos (version 26; IBM Corp.) was used. In addition to the direct and indirect effects of variables, Amos provides bootstrapped confidence intervals (95%) and *p*-values for all effects. The effects are considered significant if the confidence interval does not contain zero. Since even small relationships and differences may be statistically significant with a sample of 355, we also report on effect sizes following the guidelines provided by [36].

### 2.5. Ethics

Ethical approval for the study was granted by the Humanities and Social Sciences Ethics Committee of the University of the Western Cape (ethics reference number: HS21/3/8). Participants completed the survey anonymously and provided informed consent. Since the questionnaires focused on mental health outcomes associated with COVID-19, participants were also provided with resources for psychological counselling support in the event that their completion of the survey resulted in distress. 

## 3. Results

Prior to the main analyses, we examined gender differences, differences between those who lost a family member or colleague and those who did not, and the infected status of respondents in terms of all the study variables. We also examined the relationship between age and the study variables. There were gender differences only in terms of personal accomplishment (t = −2.07, *p* = 0.04), emotional exhaustion (t = 3.06, *p* = 0.002), and teaching satisfaction (t = −2.03, *p* = 0.04), with women reporting lower personal accomplishment, more emotional exhaustion, and less teaching satisfaction than men. However, effect size statistics showed that these effects were small (personal accomplishment: Cohen’s d = 0.26; emotional exhaustion: Cohen’s d = 0.38; teaching satisfaction: Cohen’s d = 0.26). Respondents who had lost a family member due to COVID-19 reported experiencing more role conflict than those who had not lost a family member (t = 3.32, *p* < 0.001); however, the effect size statistic indicated that it was a small effect (Cohen’s d = 0.37). There were no differences between those who lost a colleague and those who did not in terms of any of the study variables. Respondents who had tested positive for COVID-19 reported higher levels of perceived infectability than those who were not infected (t = 2.08, *p* = 0.04), and lower levels of teaching satisfaction (t = −2.66, *p* = 0.008), but in both instances, the effect was small (perceived infectability: Cohen’s d = 0.30; teaching satisfaction: 0.38). Age was positively related to personal accomplishment (r355 = 0.17, *p* = 0.001); however, the percentage of shared variance between the two variables was only 2.89% and in terms of Cohen’s guidelines this constitutes a small effect. 

The descriptive statistics, reliabilities, and intercorrelations are presented in Table 1. All questionnaires demonstrated satisfactory reliability (α and ω = 0.78–0.92), except for the GA subscale. The GA subscale had moderate but acceptable reliability (α = 0.65; ω = 0.66).

Regarding the intercorrelations, teaching satisfaction was negatively related to perceived infectability (r353 = −0.16, *p* = 0.002), role conflict (r353 = −0.19, *p* < 0.001), small association) and ambiguity (r353 = −0.37, *p* < 0.001, large association), emotional exhaustion (r353 = −0.48, *p* < 0.001, large association), and depersonalisation (r353 = −0.36, *p* < 0.001, medium association). This finding indicates that high levels of perceived infectability, role conflict and ambiguity, emotional exhaustion, and depersonalisation are associated with low teaching satisfaction. Personal accomplishment was positively related to teaching satisfaction (r353 = 0.42, *p* < 0.001, large association), which indicates that high levels of personal accomplishment are associated with high teaching satisfaction. Role conflict and ambiguity were positively related to emotional exhaustion (RC: r353 = 0.39, *p* < 0.001; RA: r353 = 0.26, *p* < 0.001, large association); however, only role ambiguity was negatively related to personal accomplishment (r353 = −0.51, *p* < 0.001, large association). This finding indicates that high levels of role conflict and ambiguity are associated with high levels of emotional exhaustion and depersonalisation, and role ambiguity is associated with low levels of personal accomplishment. Perceived infectability (r353 = 0.27, *p* < 0.001, medium association) and germ aversion (r353 = 0.11, *p* = 0.042, small association) were positively associated with emotional exhaustion; of these factors, only perceived infectability was associated with depersonalisation (r353 = 0.22, *p* < 0.001, small association). This finding indicates that high levels of perceived infectability were associated with high levels of emotional exhaustion and depersonalisation, and high levels of germ aversion were associated with high levels of emotional exhaustion.

**Figure 1:**
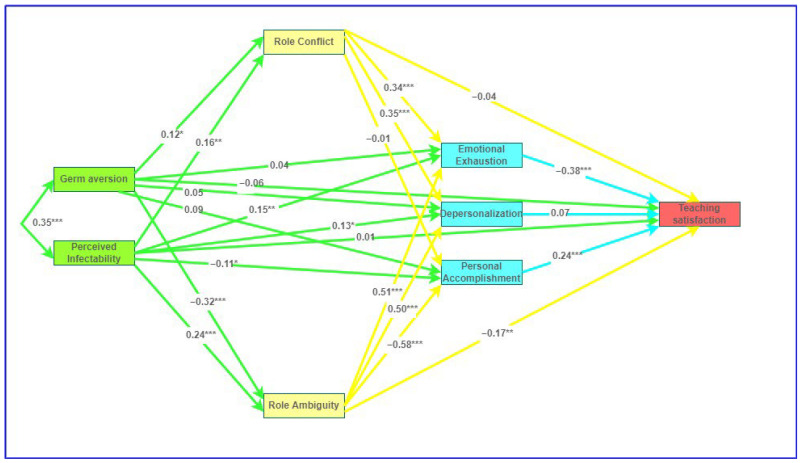
Structural equation model of the serial relationship between perceived vulnerability to disease and teacher satisfaction. Note: Regression weights are standardised. *** *p* < 0.001, ** *p* < 0.01, * *p* < 0.05.

The path analysis model that was used to examine the serial relationship between perceived vulnerability to disease, role conflict and ambiguity, burnout, and teaching satisfaction is presented in Figure 1. In this model, germ aversion and perceived infectability are predictors and teaching satisfaction is the outcome variable. The model specifies a serial relationship between the predictor and the outcome that sequentially includes role conflict and ambiguity, as well as the dimensions of burnout. 

The direct effects resulting from the path analysis model are presented in Table 2. The zero-order correlations confirmed a significant relationship between teaching satisfaction and perceived infectability, as well as role conflict; however, these relationships were non-significant when considered within the serial model (PI: β = 0.006, *p* = ns; RC: β = −0.042, *p* = ns). In the serial model, germ aversion was significantly associated with role ambiguity (β = −0.316, *p* < 0.001), role conflict (β = 0.121, *p* = 0.054), and personal accomplishment (β = 0.087, *p* = 0.067). The *p*-value in the relationship between role conflict and personal accomplishment was greater than 0.05; however, the confidence interval did not include zero. In this case, reliance was placed on the confidence interval, as suggested by [37]. Perceived infectability was significantly associated with role conflict (β = 0.243, *p* = 0.001) and role ambiguity (β = 0.157, *p* = 0.003), and it was also significantly associated with each component of burnout, namely, emotional exhaustion (β = 0.146, *p* = 0.004), depersonalisation (β = 0.122, *p* = 0.029), and personal accomplishment (β = −0.114, *p* = 0.035). Role ambiguity was significantly associated with emotional exhaustion (β = 0.268, *p* < 0.001), depersonalisation (β = 0.217, *p* < 0.001), personal accomplishment (β = −0.471, *p* < 0.001), and teaching satisfaction (β = −0.161, *p* = 0.006). However, role conflict was only significantly associated with emotional exhaustion (β = 0.346, *p* < 0.001) and depersonalisation (β = 0.328, *p* < 0.001). Finally, teaching satisfaction was significantly associated with each component of burnout, namely, emotional exhaustion (β = −0.408, *p* < 0.001), depersonalisation (β = 0.108, *p* < 0.071; significant association based on confidence interval), and personal accomplishment (β = 0.235, *p* < 0.001). 

The indirect effects are reported in Table 3. The results indicate that all study hypotheses related to emotional exhaustion and depersonalisation were supported, but not with regards to the hypotheses related to personal accomplishment. In particular, the following was found:

Hypotheses 1, 2, and 3 were supported. Germ aversion was associated with role ambiguity, which in turn was associated with all three components of burnout: emotional exhaustion (β = −0.085, *p* < 0.001), depersonalisation (β = −0.069, *p* < 0.001), and personal accomplishment (β = 0.149, *p* < 0.001).

Hypotheses 4, 5, and 6 were supported. Perceived infectability was associated with role ambiguity, which in turn was associated with emotional exhaustion (β =0.065, *p* < 0.001), depersonalisation (β = 0.053, *p* < 0.001), and personal accomplishment (β = −0.115, *p* < 0.001).

Hypotheses 7 and 8 were supported. Germ aversion was associated with role conflict, which in turn was associated with emotional exhaustion (β = 0.042, *p* = 0.039) and depersonalisation (β = 0.040, *p* = 0.039) but not personal accomplishment (β = −0.002, *p* = ns).

Hypothesis 9 was not supported. The serial relationship which involves germ aversion, role conflict, and personal accomplishment was not significant (β = −0.002, *p* = ns).

Hypotheses 10 and 11 were supported. Perceived infectability was associated with role conflict, which in turn was associated with emotional exhaustion (β = 0.054, *p* = 0.002) and depersonalisation (β = 0.051, *p* = 0.002), but not personal accomplishment (β = −0.002, *p* = ns).

Hypothesis 12 was not supported. The serial relationship involving perceived infectability, role conflict, and personal accomplishment was not significant (β = −0.002, *p* = ns).

Hypotheses 13, 14, and 15 were supported. Germ aversion was associated with increased role ambiguity, which in turn was associated with emotional exhaustion, depersonalisation, and personal accomplishment. These components of burnout were in turn associated with teaching satisfaction (EE: β = −0.085, *p* < 0.001; DP: −0.069, *p* = 0.049; PA: β = 0.149, *p* < 0.001). 

Hypotheses 16, 17, and 18 were supported. Perceived infectability was associated with increased role ambiguity, which in turn was associated with emotional exhaustion, depersonalisation, and personal accomplishment. These components of burnout were in turn associated with teaching satisfaction (EE: β = 0.065, *p* < 0.001; DP: β = 0.053, *p* = 0.040; PA: β = −0.115, *p* < 0.001). 

Hypotheses 19 and 20 were supported. Germ aversion was associated with increased role conflict, which in turn was associated with emotional exhaustion and depersonalisation. These factors were in turn associated with teaching satisfaction (EE: β = 0.042, *p* = 0.031; DP: β = 0.042, *p* = 0.051). Confidence intervals were used to determine the significant association with depersonalisation. 

Hypothesis 21 was not supported. The serial relationship involving germ aversion, role conflict, personal accomplishment, and teaching satisfaction was non-significant (β = −0.002, *p* = ns).

Hypotheses 22 and 23 were supported. Perceived infectability was associated with role conflict, which in turn was associated with emotional exhaustion and depersonalisation. These factors were in turn associated with teaching satisfaction (EE: β = 0.054, *p* < 0.001; DP: β = 0.051, *p* < 0.040). 

Hypothesis 24 was not supported. The serial relationship involving perceived infectability, role conflict, and personal accomplishment was non-significant (β = −0.002, *p* = ns).

In addition to the results related to the hypotheses, Table 3 reflects the significant role of emotional exhaustion in the relationships between role conflict and teaching satisfaction (β = −0.141, *p* < 0.001) and role ambiguity and teaching satisfaction (β = −0.109, *p* < 0.001). Depersonalisation played a significant role in the role conflict–teaching satisfaction relationship (β = 0.035, *p* < 0.059) and the role ambiguity–teaching satisfaction relationship (β = 0.023, *p* < 0.053). Confidence intervals were used to evaluate the significance of these paths. In addition, personal accomplishment played a significant role in the relationship between role ambiguity and teaching satisfaction (β = −0.111, *p* < 0.001). 

## 4. Discussion

Existing studies undertaken over the past two decades (e.g., [20,21,22,31]) have confirmed that teaching is a highly stressful profession and that schoolteachers are at significant risk of adverse mental health outcomes, particularly burnout. Studies, e.g., [38,39,40,41,42,43,44,45], have also demonstrated that teacher burnout is predictive of reduced work engagement, increased rates of depression and motivation to leave the profession, and actual teacher attrition. As previously mentioned, the JDR model [19,43] has been effectively used as a conceptual framework to understand the interplay of stressors and protective factors in influencing job satisfaction. The pandemic and its prevention measures generated new job demands for teachers, including rapidly transitioning to remote online learning, upskilling in the use of digital technology, adapting the curriculum to suit electronic delivery formats, and using information communication technology to engage with colleagues and administrators [19]. For schoolteachers in developing contexts such as South Africa who may not be familiar with online platforms, learning to use these tools can be stressful. Furthermore, in the context of the return to conventional teaching during the pandemic, as was the case in South Africa, teachers would still have to contend with new job demands that include fear of contracting the virus, using personal protective equipment at school, and monitoring adherence to prevention measures among students [13,23,24]. Many public schools in South Africa do not have the infrastructure to implement COVID-19 prevention measures (e.g., proper sanitisation facilities, running water) [46,47,48]. Hence, the demands of the job may not be matched by the presence of resources that could facilitate coping. This could potentially enhance teachers’ perceptions of their vulnerability to infection and increase their fear of COVID-19. It is against this backdrop that the current study examined the serial relationship between perceived vulnerability to disease, role stress (i.e., role conflict and ambiguity), burnout, and teaching satisfaction among a sample of South African school teachers. 

There were several important findings. First, the results confirm the relationship between role stress and burnout proposed in prior studies, e.g., [49,50,51,52]; specifically, high levels of role stress (i.e., role ambiguity and role conflict) were associated with high levels of emotional exhaustion. The COVID-19 pandemic has dramatically altered teachers’ professional identities, role expectations and increased their workloads, contributing to high levels of burnout and role stress [41,45,49]. These changes have a direct effect on teacher attrition and student learning outcomes [41]. Reference [32] argues that teachers’ primary role and professional identity is grounded in an ethos of care, which enhances their vulnerability to emotional exhaustion and role stress. High levels of stress among teachers can translate to reduced motivation and poor teaching performance, which can impact on student learning [20]. 

Second, the results provide support for the relationship between burnout and teaching satisfaction proposed in prior studies, e.g., [39,40,42]. Specifically, high levels of emotional exhaustion and low levels of personal accomplishment negatively impacted teaching satisfaction. According to the JDR model [19], the physical, psychological, social, and organisational demands of an occupation, in conjunction with the resources available to effectively perform the job, mediate emotional exhaustion and influence appraisals of personal accomplishment. COVID-19 has placed significant strain on teachers to adapt traditional face-to-face pedagogy to digital distance-learning modalities in a short amount of time with limited resources while also managing parent and student concerns, as well as the stressors of their own daily lives. These factors can precipitate increased burnout and thereby decrease job satisfaction [41,42]. 

Third, high levels of role conflict and ambiguity were associated with low levels of teaching satisfaction. COVID-19 has forced teachers to reconstruct what it means to be a teacher within the context of a pandemic [13]. This reconstruction is influenced by changing role expectations, which increase role conflict and decrease job satisfaction [45]. Coupled with the fragility of the South African education system, the act of teaching learners becomes only one facet of the job. The job demand to job resource ratio is a pervasive issue that affects most schools in South Africa [23,24]. Ultimately, the confluence of teachers’ roles and the inherent structural challenges that teachers navigate increase role stress, which negatively impacts job satisfaction.

Fourth, high levels of perceived vulnerability to disease (i.e., germ aversion and perceived infectability) were associated with high levels of role stress (i.e., role ambiguity and role conflict) and burnout, which in turn were related to low levels of teaching satisfaction. With the easing of lockdown regulations, teachers are expected to resume traditional pedagogies, which entails being physically present in small classrooms and managing large groups of students [44,45]. Given the limited personal protective resources available, these changes put teachers at an increased risk of infection, which may affect their perceptions of their own vulnerability to contracting COVID-19 and thus decrease their job satisfaction. 

Finally, no serial mediation was apparent for any of the study variables with the personal accomplishment dimension. One interpretation may be that positive evaluations of achievement in the work environment may be less susceptible to mediation effects. It is also possible that under conditions of high stress, teachers may attribute their work successes and thus feelings of personal accomplishment to their own capacities, and this may enhance their sense of self-efficacy and ability to cope under pandemic conditions. Alternatively, in the context of an unprecedented pandemic, it is probable that teachers may believe that they are doing the best that they can and this in itself, is an accomplishment. 

The study findings have implications for professional interventions. The JDR model [19] proposes that teachers can be resilient in the context of high work demands if they are offered adequate support, clear instruction, and high levels of decision-making latitude. Providing teachers with opportunities to have input on their work roles and responsibilities and clarity regarding their occupational tasks could help to reduce burnout. The provision of safety protocols in schools could also help teachers manage their COVID-19-related fears and anxieties. In addition, incorporating teacher mental health days in the school calendar and increasing access to mental health support services for teachers could help build resilience and promote job satisfaction [49]. 

The study has several limitations. The reliance on self-report measures may have restricted responses to teachers with internet access. The sample of teachers was mostly drawn from one geographic area, and future studies of samples with broader demographics are necessary to confirm the findings. Future studies could also incorporate a qualitative focus to discern teachers’ lived experiences of role conflict, ambiguity, and burnout to identify sources of resilience and coping. 

## 5. Conclusions

To the best of the authors knowledge, this represents one of the few studies to investigate the association of perceived vulnerability to disease, burnout, and teaching satisfaction. The majority of studies on the role of susceptibility to infection have focused on the role of this variable in adherence to COVID-19 safety protocols. Teachers who appraised themselves as being more vulnerable to contracting COVID-19 experienced greater role conflict and ambiguity, which in turn was associated with high levels of burnout and low satisfaction with the teaching profession. Theoretically, this study adds to the knowledge base on job resources and job demands among schoolteachers during the pandemic. It highlights that fear of COVID-19 and threat appraisals related to contracting the disease represent an additional job demand in the context of the pandemic. The study suggests that this type of job demand needs to be matched with appropriate job resources (e.g., school infrastructure and access to personal protective equipment) to enable teachers to effectively cope with the impact of the pandemic. 

## Figures and Tables

**Table 1:** Descriptive statistics, reliabilities, and intercorrelations between variables.

	1	2	3	4	5	6	7	8
1. Germ aversion	—							
2. Perceived infectability	0.35 ***	—						
3. Role conflict	0.18 **	0.21 ***	—					
4. Role ambiguity	−0.24 ***	0.13 *	0.04	—				
5. Emotional exhaustion	0.11 *	0.27 ***	0.39 ***	0.29 ***	—			
6. Depersonalisation	0.06	0.22 ***	0.37 ***	0.25 ***	0.61 ***	—		
7. Personal accomplishment	0.15 **	−0.15 **	−0.04	−0.51 ***	−0.33 ***	−0.29 ***	—	
8. Teaching satisfaction	−0.02	−0.16 **	−0.19 **	−0.38 ***	−0.49 ***	−0.26 ***	0.42 ***	—
Mean	42.9	28.7	39.4	14.7	25.0	15.2	20.0	17.3
SD	8.4	8.8	8.2	5.7	7.5	7.4	6.9	4.7
Alpha	0.65	0.78	0.83	0.83	0.94	0.85	0.84	0.87
Omega	0.66	0.78	0.83	0.83	0.94	0.86	0.84	0.87

*** *p* < 0.001, ** *p* < 0.01, * *p* < 0.05.

**Table 2:** Direct effects of predictor variables in the serial model.

Effect	Beta	SE	β	95% CI	*p*
GA → RA	−0.211	0.041	−0.316	[−0.407, −0.231]	0.001
GA → RC	0.117	0.058	0.121	[0.014, 0.207]	0.054
GA → EE	0.106	0.093	0.005	[−0.035, 0.139]	0.304
GA → DP	0.004	0.046	0.045	[−0.087, 0.092]	0.956
GA → PA	0.114	0.064	0.087	[0.008, 0.168]	0.067
GA → Satisfaction	−0.025	0.029	−0.046	[−0.126, 0.039]	0.400
PI → RA	0.156	0.036	0.243	[0.157, 0.326]	0.001
PI → RC	0.146	0.051	0.157	[0.073, 0.250]	0.003
PI → EE	0.253	0.088	0.146	[0.065, 0.229]	0.004
PI → DP	0.103	0.046	0.122	[0.032, 0.212]	0.029
PI → PA	−0.143	0.065	−0.114	[−0.196, −0.025]	0.035
PI → Satisfaction	0.003	0.026	0.006	[−0.072, 0.084]	0.892
RA → EE	0.724	0.141	0.268	[0.187, 0.351]	0.001
RA → DP	0.285	0.067	0.217	[0.132, 0.300]	0.001
RA → PA	−0.924	0.094	−0.471	[−0.546, −0.388]	0.001
RA → Satisfaction	−0.134	0.050	−0.161	[−0.266, −0.064]	0.006
RC → EE	0.643	0.096	0.346	[0.265, 0.430]	0.001
RC → DP	0.295	0.044	0.328	[0.249, 0.396]	0.001
RC → PA	−0.018	0.062	−0.014	[−0.088, 0.065]	0.757
RC → Satisfaction	−0.024	0.027	−0.042	[−0.116, 0.040]	0.419
EE → Satisfaction	−0.126	0.020	−0.408	[−0.501, −0.304]	0.001
DP → Satisfaction	0.069	0.039	0.108	[0.012, 0.209]	0.071
PA → Satisfaction	0.100	0.023	0.235	[0.148, 0.326]	0.001

Note. GA = germ aversion, PI = perceived infectability, RA = role ambiguity, RC = role conflict, EE = emotional exhaustion, DP = depersonalisation, PA = personal accomplishment, Satisfaction = teaching satisfaction.

**Table 3:** Indirect effects of perceived vulnerability and role conflict and ambiguity.

Effect	Beta	SE	β	95% CI	*p*
GA → RA → EE ^1^	−0.153	0.039	−0.085	[−0.226, −0.098]	0.001
GA → RA → DP ^2^	−0.060	0.018	−0.069	[−0.096, −0.034]	0.001
GA → RA → PA ^3^	0.195	0.043	0.149	[0.131, 0.272]	0.001
PI → RA → EE ^4^	0.113	0.033	0.065	[0.064, 0.176]	0.001
PI → RA → DP ^5^	0.044	0.016	0.053	[0.023, 0.076]	0.001
PI → RA → PA ^6^	−0.144	0.035	−0.115	[−0.207, −0.092]	0.001
GA → RC → EE ^7^	0.075	0.039	0.042	[0.014, 0.143]	0.039
GA → RC → DP ^8^	0.034	0.018	0.040	[0.007, 0.065]	0.039
GA → RC → PA ^9^	−0.002	0.008	−0.002	[−0.019, 0.008]	0.576
PI → RC → EE ^10^	0.094	0.036	0.054	[0.045, 0.163]	0.002
PI → RC → DP ^11^	0.043	0.016	0.051	[0.020, 0.075]	0.002
PI → RC → PA ^12^	−0.003	0.010	−0.002	[−0.021, 0.011]	0.681
GA → RA → EE → Satisfaction ^13^	0.019	0.006	−0.085	[0.012, 0.032]	0.001
GA → RA → DP→ Satisfaction ^14^	−0.004	0.003	−0.069	[−0.011, −0.001]	0.049
GA → RA → PA → Satisfaction ^15^	0.019	0.043	0.149	[0.011, 0.032]	0.001
PI → RA → EE → Satisfaction ^16^	−0.014	0.005	0.065	[−0.024, −0.008]	0.001
PI → RA → DP → Satisfaction ^17^	0.003	0.002	0.053	[0.001, 0.008]	0.040
PI → RA → PA → Satisfaction ^18^	−0.014	0.005	−0.115	[−0.025, −0.008]	0.001
GA → RC → EE → Satisfaction ^19^	−0.009	0.005	0.042	[−0.019, −0.002]	0.031
GA → RC → DP→ Satisfaction ^20^	0.002	0.002	0.042	[0.000, 0.007]	0.051
GA → RC → PA → Satisfaction ^21^	0.000	0.001	−0.002	[−0.002, 0.001]	0.557
PI → RC → EE → Satisfaction ^22^	−0.012	0.005	0.054	[−0.022, −0.006]	0.001
PI → RC → DP → Satisfaction ^23^	0.003	0.002	0.051	[0.001, 0.008]	0.040
PI → RC → PA → Satisfaction ^24^	0.000	0.001	−0.002	[−0.002, 0.001]	0.631
RC → EE → Satisfaction	−0.081	0.018	−0.141	[−0.115, −0.055]	0.001
RC → DP → Satisfaction	0.020	0.012	0.035	[0.003, 0.042]	0.059
RC → PA → Satisfaction	−0.002	0.006	−0.003	[−0.013, 0.008]	0.726
RA → EE → Satisfaction	−0.091	0.025	−0.109	[−0.136, −0.055]	0.001
RA → DP → Satisfaction	0.020	0.013	0.023	[0.003, 0.045]	0.053
RA → PA → Satisfaction	−0.092	0.022	−0.111	[−0.132, −0.057]	0.001

Note. ^1–24^ correspond to numbering of hypotheses. GA = germ aversion, PI = perceived infectability, RA = role ambiguity, RC = role conflict, EE = emotional exhaustion, DP = depersonalisation, PA = personal accomplishment, Satisfaction = teaching satisfaction.

## Data Availability

The datasets generated and/or analysed during the current study are available from the corresponding author upon reasonable request.
